# Unusual magneto-optical behavior induced by local dielectric variations under localized surface plasmon excitations

**DOI:** 10.1186/1556-276X-6-408

**Published:** 2011-06-02

**Authors:** Juan B González-Díaz, Antonio García-Martín, Gaspar Armelles Reig

**Affiliations:** 1 IMM-Instituto de Microelectrónica de Madrid (CNM-CSIC), Isaac Newton 8, PTM, Tres Cantos, E-28760 Madrid, Spain

## Abstract

We study the effect of global and local dielectric variations on the polarization conversion *r*_ps _response of ordered nickel nanowires embedded in an alumina matrix. When considering local changes, we observe a non-monotonous behavior of the *r*_ps_, its intensity unusually modified far beyond to what it is expected for a monotonous change of the whole refractive index of the embedding medium. This is related to the local redistribution of the electromagnetic field when a localized surface plasmon is excited. This finding may be employed to develop and improve new biosensing magnetoplasmonic devices.

## 

During the last years, a great effort has been devoted to the study of metallic nanoparticles due to their distinct optical properties with respect to that of the bulk material [1]. These differences arise mainly from their ability to uphold charge density oscillations known as localized surface plasmons (LSPs). These spatially localized modes may appear at a metal/dielectric interface, manifesting themselves as optical resonances in the transmission and reflection spectra, being their most significant feature the local enhancement of the electromagnetic (EM) field at the metal/dielectric interface [2]. The spectral position, width, and intensity of the optical resonances are extremely dependent on the size, shape, particle inter-distance, embedding environment, or material components of the nanoparticles. In a number of works, the influence of such parameters has been thoroughly studied putting forward the possibility of tailoring their optical response through the morphology of the particles [3-6].

More recently, the optical response arising from the combination of both surface plasmon resonances and magneto-optical (MO) properties that takes place in ferromagnetic nanoparticles is under intensive study. Different theoretical and experimental works [7-11] have pointed out that LSPs affect the MO response, finding an enhancement of the signal that has been usually ascribed to a pure optical effect related to the plasmonic excitation [10,12-14]. However, the MO activity defines in terms of the reflectivity coefficients as *Φ *= *r*_ps_/*r*_pp_, being *r*_ps _the polarization conversion and *r*_pp _the optical response (when the magnetic field is applied perpendicular to the sample plane). Therefore, the MO response may also be enhanced by modifying *r*_ps_. This was first shown in [11], where the authors suggested as a possible origin the strong localization of the EM field in the MO active material due to the LSP excitation. The scope of this work is to study more in detail the correspondence between the polarization conversion and the EM field under LSP excitations. To do so, we will theoretically analyze the *r*_ps _dependence to global and local dielectric changes of the surrounding media in periodic ferromagnetic nanowire arrays. We will show that the different dielectric environments affect the EM field distribution when the LSP is excited, consequently changing the spectral position and intensity of the *r*_ps _peak. Moreover, we will prove that variations of the refractive index in the close vicinity of the wires extremely affect the *r*_ps_, making its intensity much larger and/or smaller than that obtained if the whole embedding matrix is replaced. This is a consequence of the local redistribution of the EM field induced by the plasmon excitation at the metal/dielectric interface.

To investigate the influence of LSPs on the *r*_ps _response, we considered an ordered hexagonal array of nickel nanowires embedded in a dielectric matrix and oriented along the *z*-axis. The diameter of the wires was set to 80 nm, with a lattice parameter of 180 nm and a height of 15 μm (a schematic view of the model system can be seen on top of Figure 1a). The spectral dependence of the absolute value of the polarization conversion |*r*_ps_| was obtained by means of a scattering matrix method (SMM), modified to allow MO activity in the polar configuration [15]. The diagonal and off-diagonal dielectric constants of nickel were taken from [16,17], respectively, whereas the refractive index of the dielectric matrix remained energy independent. Calculations were performed for different embedding mediums (from *n *= 1.7 to *n *= 1.4), shown in Figure 1a. A peak can be observed in all the spectra, blue-shifting and increasing its intensity, as the refractive index decreases. This peak is originated by an LSP excitation in the wires, as it was pointed out in [10,11], being its spectral position related to the variation of the plasmon resonance condition introduced by the modification of the dielectric background. We also performed additional calculations replacing the hexagonal array of nanowires with an effective layer. Since the dimensions of the nanostructure are much smaller than the wavelength of light, the optical properties of the nanowires and the embedding matrix can be merged by means of an effective medium approximation (EMA) [18]. The results are shown in Figure 1b. As it can be observed, the spectra show the LSP-induced peak, but contrary to the SMM results, its intensity decreases with the refractive index.

**Figure 1:**
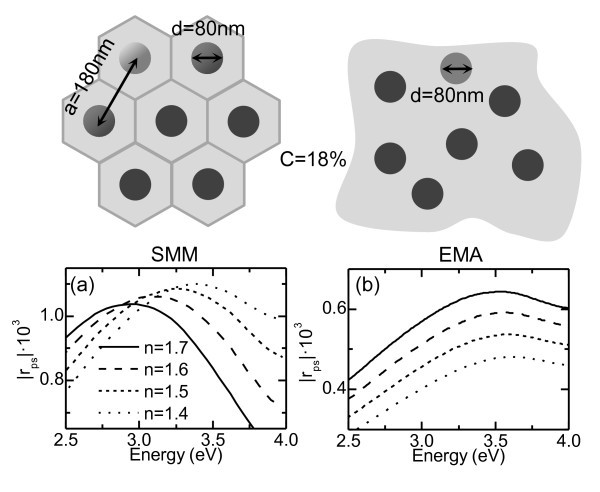
**Polarization conversion calculations**. For a system composed of nickel nanowires embedded in a dielectric medium with different refractive indexes, using (a) an SMM algorithm and (b) an EMA approximation. The schematics above show the parameters employed for each calculation. The nickel concentration in the system is the same in both calculations *C *= 18%.

The main reason why both calculations do not present similar evolutions of the polarization conversion is that the EMA approximation cannot take into account the strong increase of the EM field at the metallic nanoparticle. This can be better seen obtaining the EM field distribution within the nanowires at selected wavelengths. To do so, a 3D finite-difference time-domain (FDTD) simulation software was used (Lumerical Solutions, Inc., Vancouver, Canada), the results depicted in Figure 2 for the same parameters and refractive indexes used in the SMM calculations. The hexagons represent the unit cell showing the EM field intensity in the system at the energy where the LSP is excited. The field distribution is depicted on top of the nanostructure since its profile does not depend on the *z*-axis (just its intensity). The circle delimits the nanowire section. As it can be observed, the EM field tends to localize at the interface between the dielectric and the nanowire. When the refractive index of the matrix decreases, it appears less localized at the metal/dielectric interface, which is the expected for a plasmonic behavior. As a consequence, the EM field increases within the nanowires. Figure 2 shows this evolution with the refractive index, where we plot the average EM field spectra for different embedding matrices within the nanowires. The curves reproduce the same trend observed for the |*r*_ps_| calculations, i.e., the intensity increases as the refractive index decreases, thus pointing out that the strong relation between the polarization conversion and the amount of EM field within the nanowires induced by LSP excitations.

**Figure 2:**
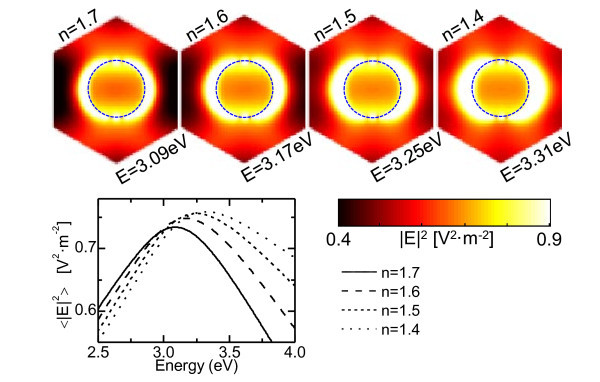
**(Graph) Theoretical spectra of the average EM field intensity within the Ni nanowires**. For the same system described in Figure 1a. The continuous, dashed, short-dashed, and dotted lines correspond to a decreasing refractive index of the embedding medium (from *n *= 1.7 to *n *= 1.4, respectively). (Top) Unit cells employed in the FDTD calculations, showing the EM field distribution in the system at the energies where the LSP is excited (maximum field concentration within the nanowire). The dashed ring delimits the nanowire section.

In this respect, the localization of the EM field at the plasmonic resonance allows studying its influence on the polarization conversion response to local dielectric changes in the dielectric matrix, which may find important applications in biosensing [19]. To do so, we considered a cylindrical shell, surrounding the nanowire with a different refractive index to that of the embedding matrix. The effects of the shell were studied for different thicknesses, from 0 nm (no shell) to 50 nm (neighboring shells in contact), and for different dielectric values: (a) *n *= 1.4 (*n *= 1.7 for the matrix) and (b) *n *= 1.7 (*n *= 1.4 for the matrix). Figure 3a, b shows the spectral position and intensity of the |*r*_ps_| peak as a function of the shell thickness for the different (a) and (b) dielectric environments (dots and circles, respectively). The black and dotted horizontal lines correspond to the values for the *n *= 1.7 and *n *= 1.4 uniform dielectric backgrounds, respectively. For both dielectric environments, the spectral position of the |*r*_ps_| peak (see Figure 3a) shifts almost linearly with the shell thickness. On the contrary, the evolution of its intensity does not appear to happen in a linear way. For example, if we restrict to the first case (a), a 5-nm shell around the wires implies a strong decrease of the intensity for the |*r*_ps_| peak. A 20-nm shell leads to the maximum decrease, and beyond this thickness, the value of |*r*_ps_| approaches gradually to that of the uniform dielectric medium. On the other hand, case (b) shows that the intensity increases above the values for the two uniform backgrounds, being the 15-nm thick shell the one that leads to the maximum |*r*_ps_|. It is worth noticing that in both cases, there is a range of shell thicknesses in which the value of |*r*_ps_| exceeds that obtained if the whole embedding matrix had the same refractive index of the shell. In particular, if we assume that replacing the whole refractive index of the matrix represents a 100% variation of the |*r*_ps_|, then the optimum shell thicknesses for cases (a) and (b) represent more than a 200% variation of the |*r*_ps_|. It is also remarkable that employing other materials presenting a larger difference in their refractive indexes might provide a much intense variation of the |*r*_ps_|. However, in our case, we have tried to remain as realistic as possible, employing refractive indexes that have already measured in the fabrication of alumina templates [20].

**Figure 3:**
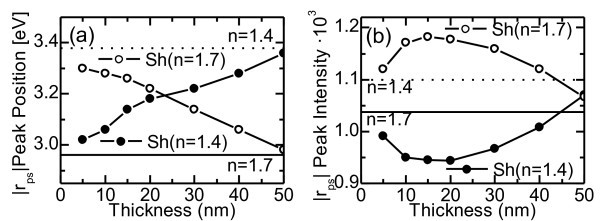
**Intensity (a) and spectral position (b) of the |*r*_ps_| peak**. As a function of a shell thickness. Dots (circles) correspond to a system composed of Ni nanowires embedded in an *n *= 1.7 (*n *= 1.4) dielectric medium and surrounded by an *n *= 1.4 (*n *= 1.7) shell. The black and dotted horizontal lines correspond to the values for the *n *= 1.7 and *n *= 1.4 uniform backgrounds respectively.

Similar to the previous analysis on global dielectric changes, these results might be a consequence of the EM field distribution within the nanowires. On top (bottom) of Figure 4, such distribution corresponding to the *n *= 1.7 (*n *= 1.4) shell is depicted at the energies where the LSP is excited. As it can be observed, when the shell presents a smaller refractive index (bottom) than the embedding matrix, the EM field within the nanowires decreases. Moreover, as the shell thickness increases, the EM field reaches a minimum that matches with that observed in the |*r*_ps_| calculations. This can be better seen in the graph of Figure 4, where we present the intensity of the average EM field within the nanowires for the (a) dielectric environments (dots). On the other hand, when the shell has a larger refractive index than the embedding matrix (b), the EM field increases within the nanowires. The average EM field for this system (circles) shows (see Figure 4) a maximum that again coincides with that obtained for the polarization conversion. This lead us to conclude that the origin of the enhanced or reduced |*r*_ps_| response in the shelled nanowires system can be ascribed to the redistribution of the EM field at the metal/dielectric interface induced by the LSP excitation, i.e., any variation of the refractive index in the vicinity of the wires affects the EM field, thus inducing a larger perturbation of the MO response.

**Figure 4:**
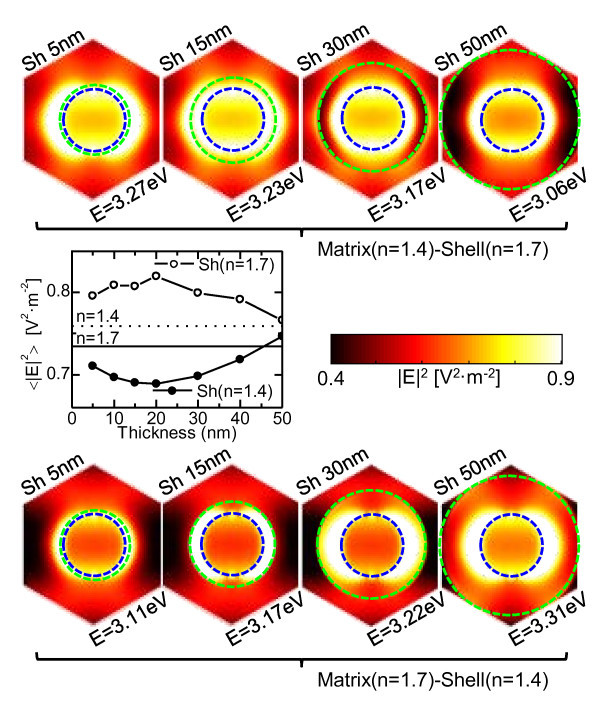
**(Top) EM field distribution of a system composed of Ni nanowires**. Embedded in an *n *= 1.4 dielectric medium and surrounded by an *n *= 1.7 shell for different thicknesses, at the energies where the LSP is excited (maximum field concentration within the nanowire). (Bottom) Same as in top but for a system composed of Ni nanowires embedded in an *n *= 1.7 dielectric medium and surrounded by an *n *= 1.4 shell. In both cases, the inner and outer dashed rings delimit the nanowire and shell sections, respectively. (Graph) Average EM field intensity within the Ni nanowires as a function of the shell thickness. The continuous and dotted lines correspond to different uniform background mediums (*n *= 1.7 and *n *= 1.4 refractive indexes, respectively), whereas circles (dots) correspond to the system described at top (bottom).

In summary, we have theoretically analyzed the relation between the LSP-induced enhancement of the EM field and the polarization conversion in hexagonally ordered ferromagnetic nanowires. We have shown that local variations of the refractive index extremely affect the |*r*_ps_| response, which is the consequence of the local EM field redistribution at the LSP resonance within the MO active material. We expect these results may find important applications in biosensing and novel magnetoplasmonic devices.

## Competing interests

The authors declare that they have no competing interests.

## Authors' contributions

JBGD carried out the theoretical simulations, AGM and GAR conceived the study. The three authors performed the data analysis, discussions of the results and wrote the manuscript.
